# CASIN exerts anti‐aging effects through RPL4 on the skin of naturally aging mice

**DOI:** 10.1111/acel.14333

**Published:** 2024-09-17

**Authors:** Yijia Zhang, Xueer Wang, Jianyuan Huang, Xinyue Zhang, Lingwei Bu, Yarui Zhang, Fengting Liang, Shenhua Wu, Min Zhang, Lu Zhang, Lin Zhang

**Affiliations:** ^1^ GDMPA Key Laboratory of key Technologies for Cosmetics Safety and Efficacy Evaluation, NMPA Key Laboratory for Safety Evaluation of Cosmetics, Guangdong Provincial Key Laboratory of Construction and Detection in Tissue Engineering, Department of Histology and Embryology, School of Basic Medical Sciences Southern Medical University Guangzhou China; ^2^ Guangdong Provincial Key Laboratory of Proteomics, Department of Pathophysiology, Key Laboratory of Mental Health of the Ministry of Education, School of Basic Medical Sciences, Center for Orthopaedic Surgery of the Third Affiliated Hospital, Guangdong‐Hong Kong‐Macao Greater Bay Area Center for Brain Science and Brain‐Inspired Intelligence Southern Medical University Guangzhou China

**Keywords:** CASIN, Cdc42, fibroblast, keratinocyte, ribosome, skin aging

## Abstract

Skin aging has been associated with the onset of various skin issues, and recent studies have identified an increase in Cdc42 activity in naturally aging mice. While previous literature has suggested that CASIN, a specific inhibitor of Cdc42 activity, may possess anti‐aging properties, its specific effects on the epidermis and dermis, as well as the underlying mechanisms in naturally aging mice, remain unclear. Our study revealed that CASIN demonstrated the ability to increase epidermal and dermal thickness, enhance dermal‐epidermal junction, and stimulate collagen and elastic fiber synthesis in 9‐, 15‐, and 24‐month‐old C57BL/6 mice in vivo. Moreover, CASIN was found to enhance the proliferation, differentiation, and colony formation and restore the cytoskeletal morphology of primary keratinocytes in naturally aging skin in vitro. Furthermore, the anti‐aging properties of CASIN on primary fibroblasts in aging mice were mediated by the ribosomal protein RPL4 using proteomic sequencing, influencing collagen synthesis and cytoskeletal morphology both in vitro and in vivo. Meanwhile, both subcutaneous injection and topical application exhibited anti‐aging effects for a duration of 21 days. Additionally, CASIN exhibited anti‐inflammatory properties, while reduced expression of RPL4 was associated with increased inflammation in the skin of naturally aging mice. Taken together, our results unveil a novel function of RPL4 in skin aging, providing a foundational basis for future investigations into ribosomal proteins. And CASIN shows promise as a potential anti‐aging agent for naturally aging mouse skin, suggesting potential applications in the field.

## Graphical abstract

CASIN increases epidermal and dermal thickness, enhances dermal‐epidermal junction (DEJ), stimulates collagen and elastic fiber synthesis, and exhibits anti‐inflammatory properties in naturally aging mice. It promotes proliferation and differentiation and restores cytoskeletal morphology in primary epidermal keratinocytes. CASIN also enhances collagen synthesis and restores cytoskeletal morphology in primary dermal fibroblasts, mediated by the ribosomal protein RPL4.

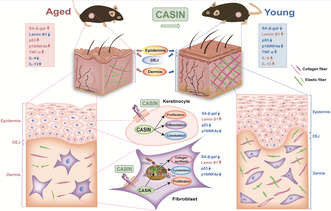

**Abbreviations**
CASIN: a specific inhibitor of Cdc42 activity
Cdc42: cell division cycle 42
DEJ: dermal‐epidermal junction
DMEM: Dulbecco's modified Eagle's medium
EdU: 5‐Ethynyl‐2′‐deoxyuridine
H&E: hematoxylin and eosin
IL‐13: Interleukin‐13
IL‐4: Interleukin‐4
K1: keratin 1
K14: keratin 14
MAPK: mitogen activated protein kinases
PBS: phosphate‐buffered saline
SDS‐PAGE: sodium dodecyl sulfate‐polyacrylamide gel electrophoresis
SEM: scanning electron microscopy
TEM: transmission electron microscopy
TEWL: transepidermal water loss
TNF‐α: Tumor necrosis factor alpha

## 1 INTRODUCTION

The skin, a vital organ responsible for maintaining homeostasis and protecting against external stimuli, undergoes aging as a natural physiological process (Gravitz, 2018). This aging process involves progressive impairments in physiological variables such as cellular redox status, immunity, and metabolism, disrupting tissue homeostasis (Kennedy et al., 2014; López‐Otín et al., 2023). Skin aging, a manifestation of organ aging in humans, results from the cumulative impact of these factors, leading to thinning of the epidermis and dermis, dermal‐epidermal junction (DEJ), collagen and elastin degradation, and compromised barrier function (Csekes & Rackova, 2021; Kohl et al., 2011). The consequences include dryness, diminished elasticity, laxity, and the formation of wrinkles, impacting both aesthetics and health (Griffiths et al., 2023).

Cell division cycle 42 (Cdc42) is a pivotal constituent of the Rho family of small G proteins. It is now understood that Cdc42 is present in two distinct states in vivo, namely the active GTP‐binding state and the inactive GDP‐binding state, regulated by GEF and GAP proteins (Huang et al., 2022). The genetic elimination of Cdc42GAP in mice, termed Cdc42GAP knockout, was found to upregulate Cdc42‐GTP levels in all tissues, contributing to age‐related pathologies such as reduced life expectancy, impaired wound healing, muscle wasting, atherosclerosis, osteoporosis, cancer, diabetes, cardiovascular disorders, and neurodegenerative conditions (Zhu, Xiao, et al., 2023). This prompts the question of whether suppressing Cdc42 activity could potentially delay skin aging (Murphy et al., 2021).

A comprehensive examination of existing literature has led us to identify a small‐molecule inhibitor, CASIN, designed for Cdc42, which effectively decreased free radical oxygen (ROS), p16INK4a, and F‐actin levels, as well as the phosphorylation of ERK1/2 and JNK in aged rat mesenchymal stem cells (Umbayev et al., 2018). Meanwhile, recent research findings indicated that systematic administration of CASIN significantly extended the average and maximum lifespan of elderly (75‐week‐old) female C57BL/6 mice over four consecutive days (Florian et al., 2020). Notably, the treatment of elderly mice with CASIN could act on hair‐follicle stem cells to induce hair regeneration and increase the percentage of anagen skin area (Tiwari et al., 2021). Despite these promising results, there is a notable lack of pertinent data on the impact of small‐molecule inhibitors targeting Cdc42 activity, specifically on keratinocytes and fibroblasts (Florian et al., 2012; Liu et al., 2019).

Alterations in protein synthesis rate and ribosomal function play pivotal roles in various molecular mechanisms associated with aging (Jiao et al., 2023). Ribosomes, responsible for intracellular protein synthesis, primarily facilitate this process by utilizing mRNA as templates and amino acids as fundamental constituents (Bassler & Hurt, 2019). Extensive research has demonstrated that ribosomes exert influence over the rate of protein synthesis and influence crucial cellular processes such as cell proliferation, differentiation, apoptosis, and transformation (Jiao et al., 2023). Disturbances in ribosome biogenesis, degradation, or changes in constituent expression can have significant consequences, potentially leading to outcomes like cell cycle arrest, aging, apoptosis, and the development of cancer or age‐related degenerative diseases. This study aimed to examine potential adverse outcomes arising from specific interventions related to ribosomal organisms (Kasselimi et al., 2022; Takada & Kurisaki, 2015).

The primary goal of our investigation was to determine the optimal concentration, duration, and biological function of CASIN to elicit anti‐aging effects in the epidermis and dermis of naturally aged mice. Meanwhile, the mechanism behind CASIN's anti‐aging properties was explored through proteomic sequencing.

## 2 MATERIALS AND METHODS

### 2.1 Experimental animals and ethics

In this study, C57BL/6 female mice (weighing 25–30 g each) of three age groups were utilized: 9 months old, 15 months old, and 24 months old. The mice adhered to regular feeding and drinking schedules, underwent frequent bedding changes, and followed a 12‐h day/night cycle. Procured from the Experimental Animal Center of Southern Medical University under Animal Production License No. SCXK (Yue) 2016–0041, these mice were handled by experiment operators possessing an animal experiment operation license (number: 202204035). All procedures were approved by the Animal Care and Use Committee of Southern Medical University (ethical review number: SMUL2022207).

### 2.2 H&E, MASSON, and elastic fiber staining

Following CASIN (Sigma, SML1253, USA) treatment, mice were euthanized on days 7, 14, and 21. Randomly distributed circles (four or two) with a 1 cm diameter were placed symmetrically on the mouse backs. Skin tissue underwent fixation in a 4% paraformaldehyde solution (PFA) for 5–7 days, followed by dehydration in an ethanol gradient, xylene translucency, wax immersion, parafilm embedding, and 5 μm sectioning. Standard procedures were employed for Hematoxylin and Eosin (H&E), Masson's trichrome stain (Maixin‐Bio, MST‐8004, China), and elastic fiber staining (Solarbio, G1593, China). The epidermal thickness, DEJ convolution(fold), dermal thickness, collagen area, collagen density, and elastic fibers area were detected by ImageJ software.

### 2.3 Immunohistochemical staining

Similar to H&E staining, 3% H_2_O_2_ incubation deactivated endogenous peroxidase, and sodium citrate antigen repair solution was used for antigen repair. A 5% BSA blocking solution with 10% goat serum was applied for 2 h. Cdc42 GTP (mouse; 1:100, NewEastBio, 26,905, USA), Cdc42 (rabbit; 1:200, Abcam, ab187643, UK), K14 (rabbit; 1:200, Abcam, ab7800, UK), Ki67 (rabbit; 1:200, Abcam, ab15580, UK), K1 (rabbit; 1:200, Abcam, ab185629, UK), Lamin B1 (rabbit; 1:300, Abcam, ab133741, UK), p16INK4a (rabbit; 1:200, Invitrogen, PA1‐30670, USA), p53 (rabbit; 1:200, Invitrogen, PA5‐27822, USA) and TNF‐α (rabbit; 1:300, Proteintech, 17,590‐1‐AP, USA), IL‐4 (mouse; 1:100, Proteintech, 66,142‐1‐Ig, USA) and IL‐13 (rabbit; 1:200, Bioss, bs‐0560R, China) were incubated overnight at 4 degrees. Secondary antibody (ZSGB‐BIO, PV‐6001, China) incubation followed at room temperature for 1 h. Diaminobenzidine (DAB) (Boster, AR1022, China) measured peroxidase activity, and Image Pro Plus was used to assess the mean number of positive cells, mean density, and average optical density.

### 2.4 Measurement of skin‐related instruments

High‐definition images of the skin surface were captured using DermoGenius ultra‐polarized dermoscopy (Germany). The AF200 AquaFlux™ transdermal dehydration measurement instrument (UK) assessed skin transepidermal water loss (TEWL). The DUB skin scanner (Germany), a high‐frequency ultrasound imaging system, was employed for skin ultrasound imaging and dermal density measurement. Skin elasticity, pigment, and moisture levels were detected using the MC750 German CK Multi Skin Test Center (Germany).

### 2.5 Scanning electron microscopy and transmission electron microscopy

The skin was quickly immersed in cold PBS upon removal to eliminate subcutaneous tissue and blood vessels. A small piece was promptly placed into an EP tube containing 2.5% glutaraldehyde for fixation. Subsequently, the samples underwent drying by spray and were subjected to analysis using scanning electron microscopy (Hitachi Science Systems, Ltd., Japan). Images were captured using a transmission electron microscope (Hitachi Science Systems, Ltd., Japan) after staining the sections.

### 2.6 Primary culture of epidermal keratinocytes

C57BL/6 female mice aged between 9 and 15 months were selected. The hair from their back skin was removed, and disinfection was performed by submerging them in a solution containing 75% ethanol and iodophor. Following the removal of the entire layer of skin from the mouse's back, the dermis and epidermis were spread in a culture dish after cleaning in D‐PBS. The prepared neutral protease (DispaseII, Roche, 04942078001, Switzerland) was gently added, and digestion occurred for 20 h at 4°C. Post‐digestion, the epidermis was gently separated from the dermis, filtered, and centrifuged at 1000 rpm for 5 min. The separated epidermis was reconstituted in KBM culture media (Lonza, CC‐3108, Switzerland), followed by cell counting and seeding on a plate.

### 2.7 Primary culture of dermal fibroblasts

After completely chopping the divided dermis, the mixture was filtered, and centrifugation at 1000 rpm for 5 min followed. The resuspended material was placed in a low‐sugar medium (Gibco, C11885500BT, USA) containing 10% FBS (Gibco, USA) after centrifugation. Cells were inoculated into a 6‐cm cell culture dish, with P3‐P4 cells used for subsequent experiments.

### 2.8 Live and death staining

Live‐death staining (AM/PI) (BestBio, BB‐4126, China) utilized a dual‐color fluorescent staining method where the AM stained live cells in green and PI stained dead cells in red. It was employed to assess the toxicity of drugs based on various CASIN concentrations (0 μM, 0.25 μM, 0.5 μM, 1 μM, 2 μM, and 4 μM). A 24‐well plate was added to the cultivated cells for 24 h. The control group consisted of a culture medium devoid of growth factors and serum. Three wells were repeated for each concentration. Working solutions A (Calcein AM) and B (PI) for staining were added sequentially. Incubation in the dark at room temperature or 37°C occurred for 30 min. After washing the cells with PBS, pictures were taken under a fluorescence microscope (Leica, DM40008, Germany).

### 2.9 EdU cell proliferation experiment

Primary keratinocytes from adult mice were cultured for 9–12 days. In a 24‐well plate, adult mouse primary fibroblasts were grown at a density of roughly 70%. After 6 h of starvation, CASIN at a concentration of 0.5 μM was added to a 24‐well plate for 24 h. The control group consisted of a culture medium without growth factors or serum. Three wells were repeated for each concentration. The EdU reagent kit (RiboBio, C10310‐3, China) was added, with a solution (1000 ×) used to incubate keratinocytes for 3 h and fibroblasts for 12 h. Following 4% PFA fixation (GBCBIO, G0528, China), EdU staining was carried out, and photos were taken using fluorescence microscopy.

### 2.10 Cytoskeleton staining

Cells were fixed with 4% PFA, and a 2 mg/mL glycine solution was used to remove any extra aldehyde groups. For incubation, 0.5% Triton X‐100 (V900502, VETEC, USA) penetrant was added. After cleaning the cells with PBS, the pre‐prepared ghost peptide staining solution (Beyotime, C0265, China) was added, avoiding light, and staining occurred at room temperature for 30 min. The anti‐fluorescent quenching agent was used to seal the film after Hoechst staining (1:1000; Invitrogen, H1398, USA) of the nucleus, and pictures were taken with an upright fluorescence microscope.

### 2.11 SA‐β‐gel staining

According to SA‐β‐Gel staining instructions (CST, 23833S, USA), cells were applied to 1× Fixative Solution Fixation, and preparation of Galactosidase Staining Solution involved combining 930 μL 1 × Staining Solution, 10 μL 100 × Solution A, 10 μL 100 × Solution B, and 50 μL 20 mg/mL X‐gal stock solution for 1 mL. Strict pH control at 6.0 was observed. Overnight incubation at 37°C was recommended, with the duration adjusted based on staining progress. Positive staining was used to observe the appearance of blue coloration.

### 2.12 Crystal violet staining

After fixing cells with 4% PFA, washing with PBS, and adding crystal violet staining solution (Beyotime, C0121, China) directly to the well plate for 10 minutes, cells were stained. Digital photography documented the growth of cell colonies.

### 2.13 Cellular immunofluorescence staining

Cells were fixed with 4% PFA, and 0.5% Triton X‐100 penetrant was used for incubation. After cleaning with PBS, cells were encapsulated in a 5% BSA‐blocking solution with 10% goat serum (Beyotime, C0265, China) for 2 h. The first antibody was incubated at 4 degrees Celsius overnight, while the second antibody was incubated at room temperature in the dark for 1 h. After staining the nucleus with Hoechst 33342 (1:1000, Sigma, 14,533, USA), sealing with an anti‐fluorescence quenching agent occurred, and pictures were taken with an upright fluorescent microscope.

### 2.14 Western blot

Proteins were extracted from skin tissue, primary keratinocytes, and dermal fibroblasts. RIPA lysis (Beyotime, P0013, China), protein concentration measurement using the BCA technique (Beyotime, P0010, China), and addition of the proper quantity of 5 × Loading buffer (Beyotime, P0015L, China) were performed. Proteins were boiled in 95°C boiling water for 10 min, with a protein sample concentration per pore of 30 mg and a 10% polyacrylamide gel (SDS‐PAGE, epizyme, PG112, China) used. Following electrophoresis, the membrane was transferred to a PVDF membrane (Millipore, IPVH00010, USA). The Lamin B1 (rabbit; 1:1000, Abcam, ab133741, UK), p16INK4a (rabbit; 1:1000, Invitrogen, PA1‐30670, USA), p53 (rabbit; 1:1000, Invitrogen, PA5‐27822, USA), p21 (rabbit; 1:1000, Abcam, ab188224, UK), Col I (rabbit; 1:500, Proteintech, 14,695‐1‐AP, USA), and RPL4 (rabbit; 1:1000, Proteintech, 11,302‐1‐AP, USA) were sealed and then incubated at 4°C overnight. After washing the TBST film, exposure occurred using an exposure meter after the secondary antibody (1:2000; Cwbio, CW0156S, China) was incubated at room temperature (Bio‐Rad) for an hour. ImageJ software was used to identify the grayscale values of the bands and analyze the relative expression level.

### 2.15 Proteomics

We used the 4D‐Lable‐Free proteomic quantitative study (PTM BIO, China) to detect the samples, which were divided into four groups, namely WT9‐N, WT9‐C, WT15‐N, and WT15‐C. The Mfuzz approach, which could conduct enrichment analysis of GO functions, KEGG pathways, and associated protein domains, was used to link the interaction between proteins and clusters for cluster analysis on the aforementioned four groups of sample data.

### 2.16 Small interfering RNA interference experiment

The P3 generation of primary fibroblasts from the back skin of 9‐month‐old mice was inoculated into a 24‐well plate and allowed to starve for 8 h after reaching about 70% cell density. According to the manufacturer's instructions, 50 nM of each siRNA (RiboBio, P202209190034, China), Lipofectamine 2000 (Invitrogen, 11,668,019, USA) and Opti‐MEM™ (Gibco, 31,985,070, USA) were utilized for siRNA transfection. In the tests, siRPL4‐001, siRPL4‐002, and siRPL4‐003, or NC, were transfected into primary fibroblasts for 8 h. Then, cell culture continued for 48 h, and Western blotting was used to confirm the siRNA's effectiveness at being transfected.

### 2.17 Subcutaneous injection of lentivirus and preparation of frozen sections

The pClinti‐U6‐shRNA (NC)—CMV‐EGFP‐WPRE (OBiO, GL404NC, China) and the pClinti‐U6‐shRNA (RPL4)—CMV‐EGFP‐WPRE (OBiO, Y24188, China) with green fluorescent markers were subcutaneously injected into two symmetrical circles with a diameter of 1 cm on the back skin of mice. The titer was 1 × 10^6^ TU/mL, and the volume was 0.2 mL. After removing the skin on the seventh day, parts of it were frozen. Simultaneously, frozen sections of skin tissues from various regions of the mice's backs belonging to the control group were prepared. After lentivirus infection of the skin, the nucleus was stained with Hoechst cell  staining solution (1:1000; Invitrogen, H1398, USA), and fluorescence expression was observed.

### 2.18 Statistical analysis

Statistical analysis was conducted using SPSS 22 (SPSS, Inc., USA). The *t*‐test compared results between two groups, while one‐way ANOVA analyzed various results of multiple groups. Standard deviations (SD) expressed the data, with statistical significance defined as *p* < 0.05.

## 3 RESULTS

### 3.1 The anti‐aging effects of subcutaneous injection of CASIN in naturally aging mouse skin

**FIGURE 1:**
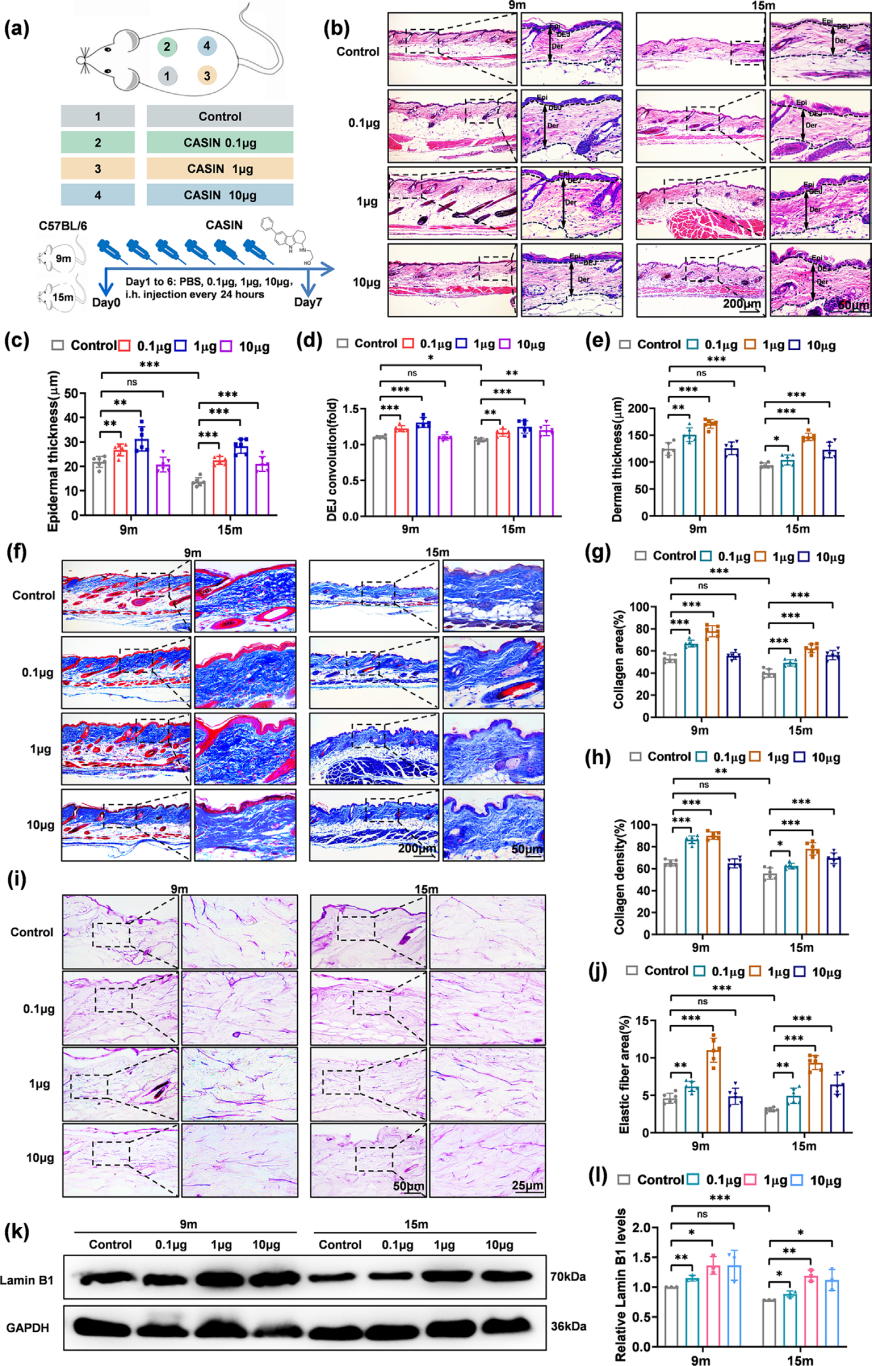
The anti‐aging effect of subcutaneous injection of CASIN in the skin of naturally aging mice. (a) Animal Pattern Diagram and experimental treatment of naturally aging mice at 9 and 15 months. Four evenly spaced, symmetrical circles with a diameter of 1 cm were randomly applied to the backs of mice. The mice were then divided into groups: Control (PBS), CASIN 0.1 μg, 1 μg, and 10 μg. The solvent used in this study was a sterile PBS solution with a volume of 200 μL. (b–e) H&E staining and statistics of epidermal thickness, DEJ convolution(fold), and dermal thickness, *n* = 6. (f–h) MASSON staining and statistics of collagen area and collagen density, *n* = 6. (i, j) Elastic fiber staining and statistics of elastic fiber area, *n* = 6. (k, l) The Western blot of Lamin B1 expression, *n* = 3; (scale bar = 200, 50, 25 μm). All error bars indicate SD. *p<0.05, **p<0.01, ***p<0.001.

Subcutaneous injections were administered continuously for 6 days, every 24 h, before removing the skin (Figure 1a). H&E staining results revealed that the epidermal and dermal thickness and DEJ decreased in 15‐month‐old mice compared with 9‐month‐old mice. On the other hand, in naturally aged mice at 9 and 15 months, the thickness of the epidermis and dermis and DEJ convolution were increased by CASIN at 0.1 μg and 1 μg, respectively (Figure 1b–e). Additionally, natural aging also results in a decrease in collagen density and area. CASIN at 0.1 μg and 1 μg significantly stimulated collagen synthesis in the dermis, leading to a statistically significant increase in collagen area and density, accompanied by a more compact and organized arrangement (Figure 1f–h). Furthermore, the presence of elastic fibers decreased in the dermis during aging. CASIN demonstrated the potential to enhance elastic fibers at concentrations of 0.1 μg and 1 μg, with the most pronounced effect observed at 1 μg (Figure 1i,j). Western blot analysis confirmed that expression of Lamin B1 decreased with aging, while there was an upregulation of Lamin B1 expression in the skin of mice following CASIN treatment at 0.1 μg and 1 μg (Figure 1k,l). Immunohistochemical analysis indicated that mice aged 15 months exhibited higher levels of Cdc42 GTP/Cdc42 expression compared to those aged 9 months. Furthermore, the expression of Cdc42 GTP/Cdc42 decreased in both 9‐month‐old and 15‐month‐old mice following CASIN therapy, with the reduction being more pronounced at 1 μg (Figure S1a,b). When CASIN was at 10 μg, there was no significant statistical significance in the above results compared to the control group in 9‐month‐old mice (Figure 1b–l). These findings suggest that the subcutaneous injection of CASIN at 1 μg yielded a significant anti‐aging effect on the skin of middle‐aged and old mice.

### 3.2 CASIN promotes epidermis anti‐aging in naturally aging mice

**FIGURE 2:**
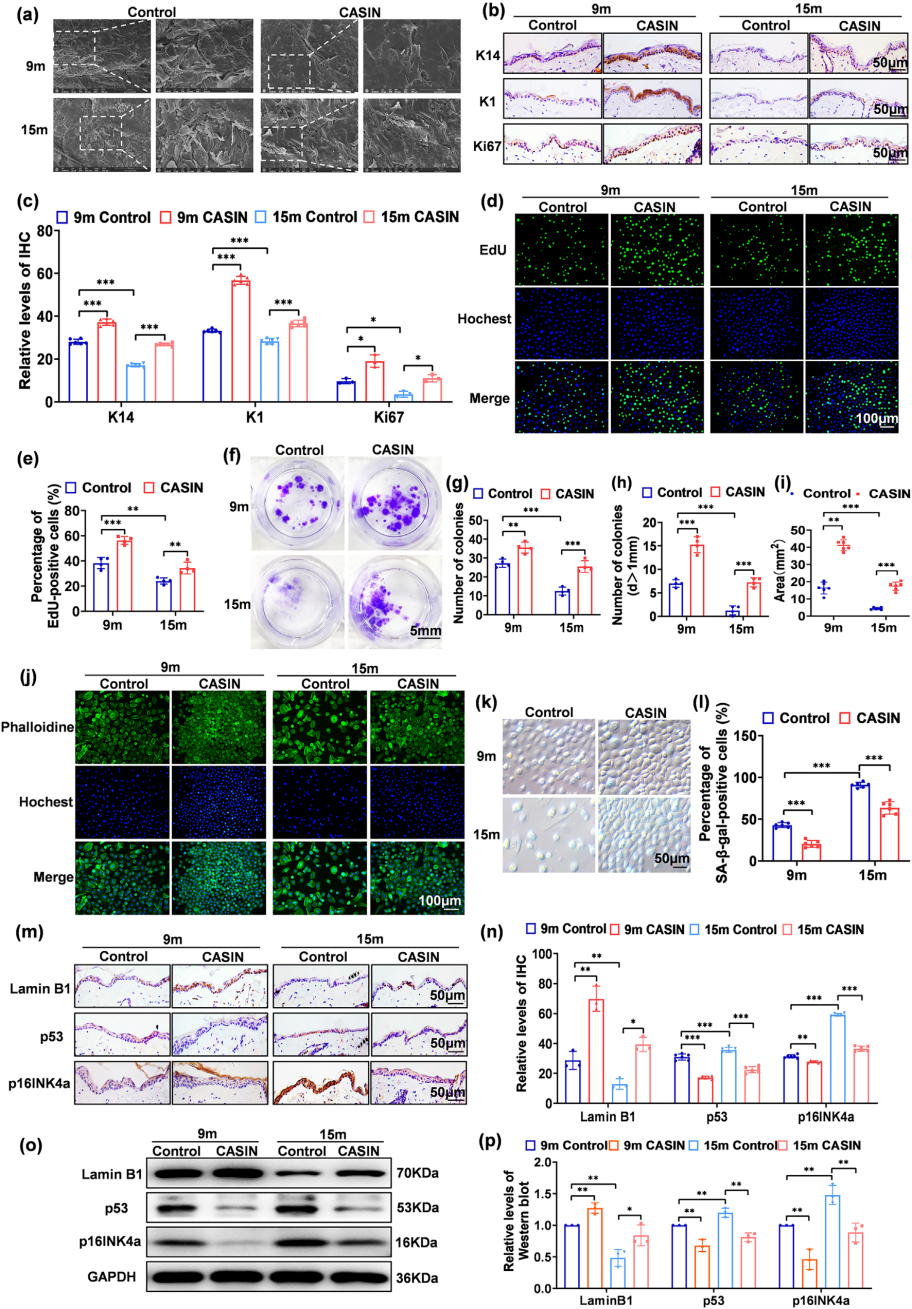
CASIN promotes the epidermal anti‐aging of naturally aging mouse skin. (a) The scanning electron microscope in the epidermis. (b, c) Immunohistochemical staining and statistics of relative levels of K14, K1, and Ki67 in the epidermis, *n* = 6. (d, e) Images of EdU positive proliferating cells and statistics of proliferation rate of primary keratinocytes, *n* = 4. (f–i) Cell colony formation by crystal purple staining and statistics of cell colony count, larger cell colonies (d > 1 mm) and colony area (mm^2^) of primary keratinocytes, *n* = 4, 6. (j) Phalloidine staining of the skeletal morphology of primary keratinocytes. (k, l) SA‐β‐gal staining and statistics of positive rate of primary keratinocytes, *n* = 6. (m, n) Immunohistochemical staining and statistics of relative levels of Lamin B1, p16INK4a, and p53 in the epidermis, *n* = 3, *n* = 6. (o, p) The Western blot detection of the anti‐aging effect of CASIN and relative expression of Lamin B1, p53, and p16INK4a of primary keratinocytes, *n* = 3; (scale bar = 100, 50 μm, 5 mm). All error bars indicate SD. *p<0.05, **p<0.01, ***p<0.001.

The natural aging process is characterized by thinning of the epidermis and a decline in keratinocyte function (Lorencini et al., 2014; Wang et al., 2020). Scanning electron microscope findings indicated that there was a noticeable increase in both the irregularity of texture arrangement and the number of wrinkles on the skin surface of natural aging mice. In contrast, the skin surfaces of 9‐month‐old and 15‐month‐old mice treated with CASIN displayed a flat appearance and exhibited fewer wrinkles and a more compact texture arrangement (Figure 2a).

Immunohistochemical analysis showed that the expression of K14, K1, and Ki67 decreased as mice naturally matured. Conversely, in the epidermis of 9 and 15‐month‐old mice treated with CASIN, there was an increase in the expression of K14, K1, and Ki67 (Figure 2b,c). Additionally, CASIN applied to primary keratinocytes derived from mouse skin demonstrated that concentrations equal to or exceeding 2 μM resulted in cell death, while concentrations equal to or below 1 μM had no impact on cell status (Figure S2a). Furthermore, EdU staining revealed that a concentration of CASIN at 0.5 μM could enhance cell proliferation (Figure S2b,c, Figure 2d,e). The rate of colony growth and fusion was observed to be slower in primary keratinocytes due to natural aging. However, in the back skin of mice aged 9 and 15 months, the area and number of primary keratinocyte colonies increased, along with an increase in the number of larger colonies (d > 1 mm), following CASIN therapy. This finding suggests that CASIN can potentially enhance the formation of primary keratinocyte colonies (Figure 2f–i). These findings indicate that CASIN can effectively stimulate the proliferation and differentiation of skin epidermal cells in naturally aging mice.

As individuals age, cellular changes associated with aging, such as alterations in cell volume and irregular cell morphology, often result in the presence of larger, “egg‐like” cells. However, after undergoing CASIN therapy, keratinocytes exhibited a reduction in the production of irregularly shaped cells, primarily adopting a standard paving stone morphology. The aforementioned findings suggest that CASIN treatment has the potential to reverse the cytoskeletal shape of primary keratinocytes in naturally aging mice (Figure 2j).

The process of natural aging is characterized by an increased positive rate of SA‐β‐gal staining, decreased Lamin B1 expression, and upregulated p16INK4a and p53 expression. The positive rate of SA‐β‐gal staining in the primary keratinocytes of these mice decreased with CASIN treatment (Figure 2k,l). Additionally, immunohistochemical and western blot revealed that CASIN treatment in 9 and 15‐month‐old mice resulted in an increase in the expression of Lamin B1 in the epidermis and primary keratinocytes, while the expression of p16INK4a and p53 decreased (Figure 2m–p). These findings suggest that CASIN exhibits anti‐aging effects in primary keratinocytes and the epidermis.

### 3.3 CASIN promotes dermis anti‐aging in naturally aging mice

**FIGURE 3:**
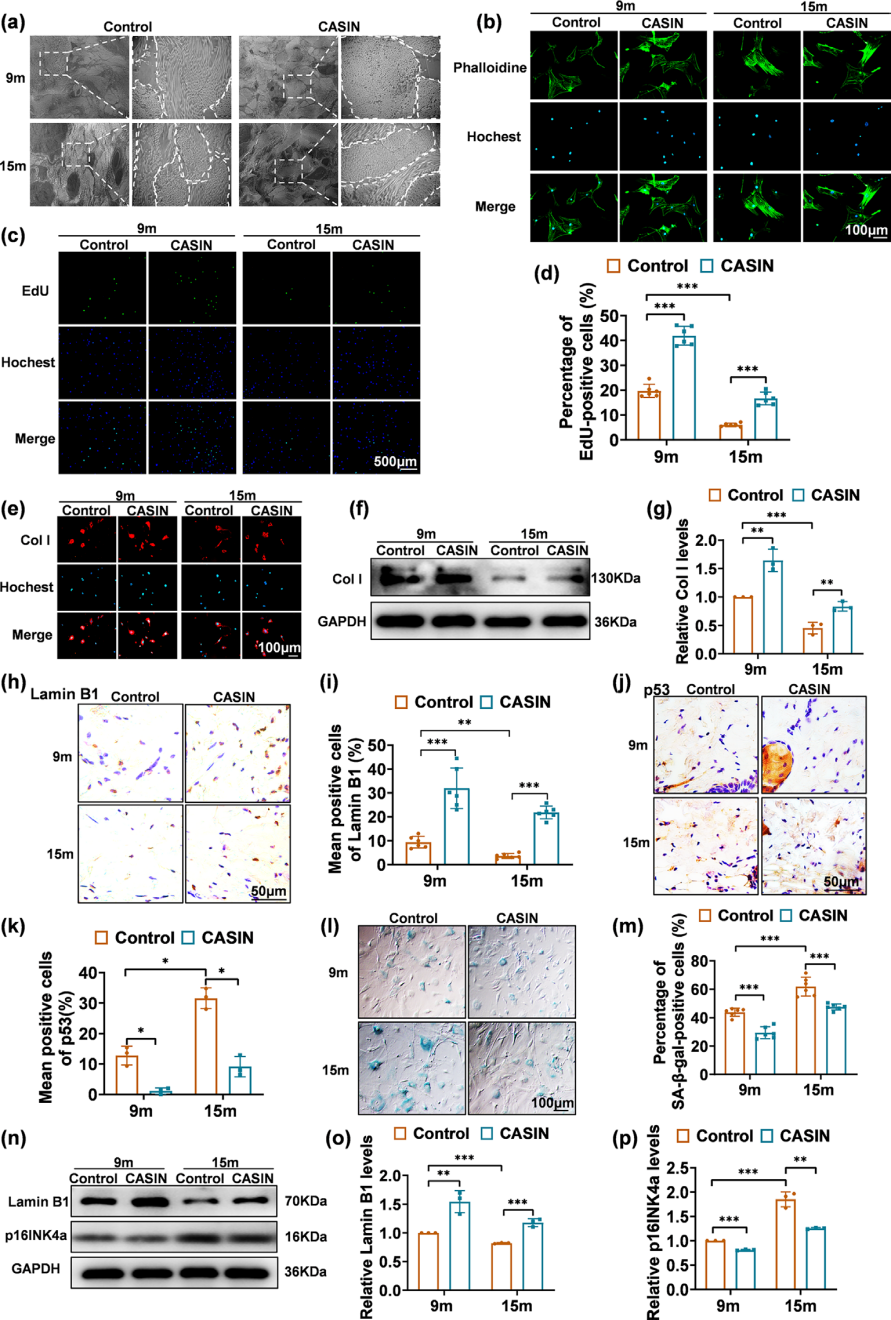
CASIN promotes the dermal anti‐aging of naturally aging mouse skin. (a) Transmission electron microscopy of the transverse section of the dermis. (b) Phalloidine staining of the skeletal morphology of fibroblast. (c, d) Images of EdU positive proliferating cells and statistics of proliferation rate, *n* = 4. (e–g) Immunofluorescence **staining,** Western blot detection and relative expression of Col I; *n* = 3. (h–k) Immunohistochemical staining and statistics of relative levels of Lamin B1 and p53, *n* = 3, *n* = 6. (l, m) SA‐β‐gal staining and statistics of positive rate, *n* = 6. (n–p) The Western blot detection of the anti‐aging effect of CASIN and relative expression of Lamin B1 and p16INK4a, *n* = 3; (scale bar = 500, 100, 50 μm). All error bars indicate SD. *p<0.05, **p<0.01, ***p<0.001.

The aging effect in the dermis of naturally aged mice is characterized by the degradation of collagen and elastic fibers, along with diminished fibroblast function (Heinz, 2021; Schmelzer & Duca, 2022; Wlaschek et al., 2021). According to transmission electron microscopy, the quantity of collagen fiber bundles naturally increases, but each bundle undergoes shrinkage, varying diameters of collagen fibrils, and more sparse distribution with age. CASIN therapy induces a thickening and enlargement of collagen fiber bundles in the mouse dermis, accompanied by a corresponding increase in the thickness and enhanced organization of the collagen fibrils encircling these bundles (Figure 3a).

Live/death staining of primary fibroblasts from mouse skin indicated that concentrations of CASIN equal to or greater than 2 μM resulted in cell death, while concentrations equal to or less than 1 μM yielded no impact on cell status (Figure S3a). Analysis of aging markers through Western Blot revealed that the optimal concentration of CASIN in primary fibroblasts was 0.5 μM (Figure S3b–d). Furthermore, the morphology of primary fibroblasts became more irregular with age. However, treatment of nine‐month‐old mouse fibroblasts with CASIN improved their form and skeletal morphology. The volume of mouse fibroblasts at 15 months of age exhibited a decrease following CASIN therapy, and their morphology displayed a tendency towards regularity (Figure 3b). EdU staining revealed that CASIN could induce primary fibroblast proliferation in mice undergoing natural aging (Figure 3c,d). Immunofluorescence and Western Blot analyses demonstrated a decline in Col I expression in fibroblasts during the process of naturally aging. However, following CASIN treatment, Col I expression exhibited an increase at 9 and 15 months (Figure 3e–g). These findings indicate that CASIN could effectively reverse the cytoskeletal shape and stimulate the proliferation and Col I of skin fibroblasts in naturally aging mice.

The immunohistochemistry data exhibited that the expression of Lamin B1 increased and P53 expression decreased in the dermis of mice aged 9 and 15 months following CASIN therapy, as depicted in Figure 3h–k. Additionally, the positive rate of SA‐β‐gal staining in primary fibroblasts decreased after CASIN therapy (Figure 3l,m). Furthermore, CASIN treatment resulted in a decrease in p16INK4a expression and an increase in Lamin B1 expression in primary fibroblasts, as observed through western blot analysis (Figure 3n–p). Based on these findings, it can be inferred that CASIN yields anti‐aging effects on the dermis of mice undergoing natural aging.

### 3.4 CASIN can exert skin anti‐aging effects through ribosomal proteins

**FIGURE 4:**
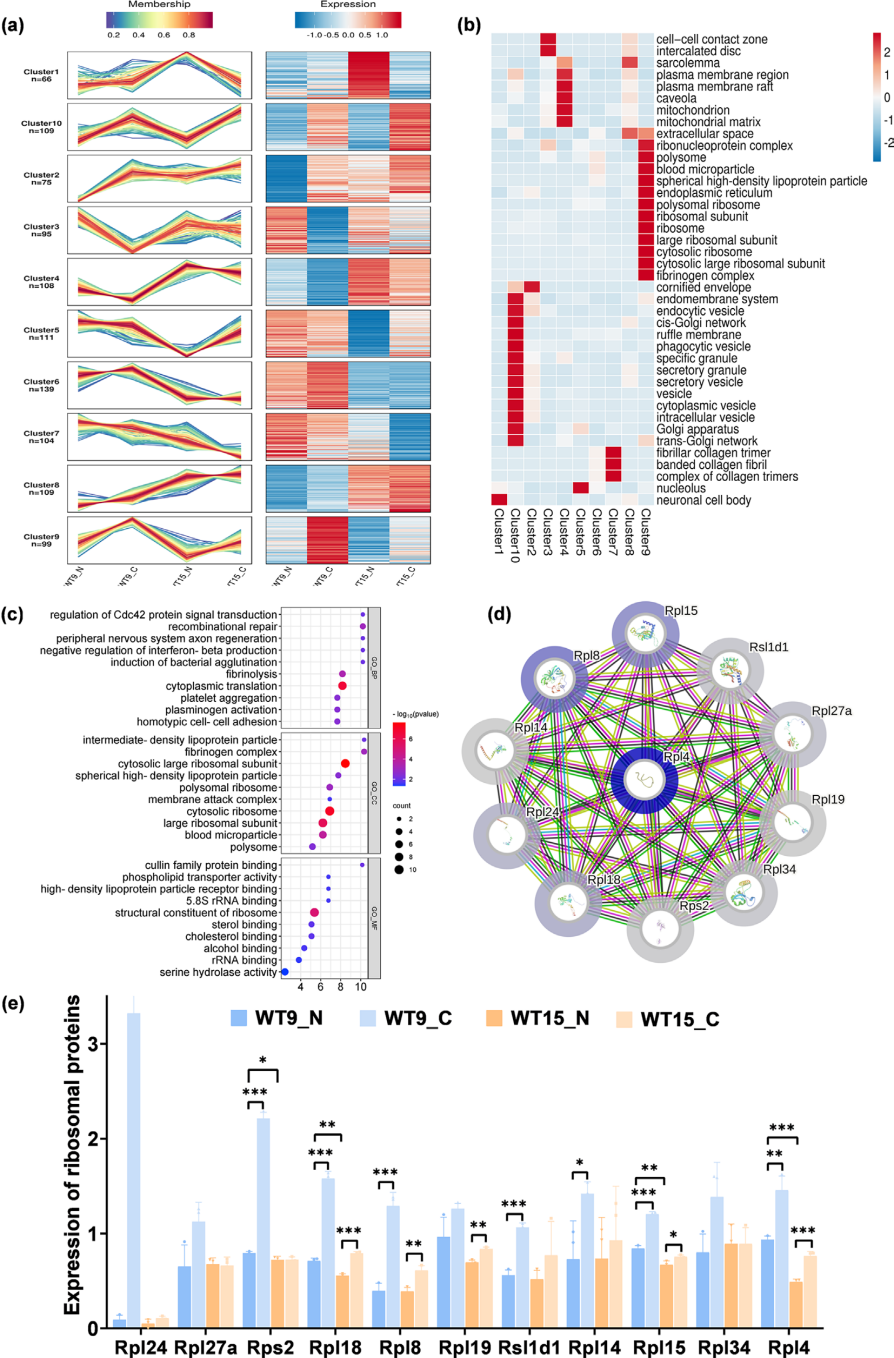
CASIN can exert skin anti‐aging effects through ribosomal proteins. (a) Mfuzz expression pattern clustering diagram of the 10 Clusters. (b) Cluster heat map of GO‐CC biological function enrichment of the 10 Clusters. (c) GO analysis was performed on the Top 10 results of Cluster 9, selected based on descending Fold Enrichment, a *p*‐value less than 0.05, and statistically significant differences for biological processes (BP), cellular components (CC), and molecular functions (MF). (d) PPI protein interaction network analysis of 11 proteins with ribosome‐related functions between WT9‐N and WT15‐N groups of Cluster 9 via the String database. (e) Statistics of the expression of 11 proteins with ribosome‐related functions in WT9‐N, WT9‐C, WT15‐Nand WT15‐C groups of Cluster 9. All error bars indicate SD. *p<0.05, **p<0.01, ***p<0.001.

In order to investigate the molecular mechanism of CASIN's role in the anti‐aging process of skin, 4D‐Lable‐Free proteomics was used for a quantitative study. A total of 6672 proteins were identified, and 1015 proteins were divided into 10 clusters for Mfuzz expression pattern cluster analysis. A significant decrease in the expression levels of proteins associated with Cluster 9 was observed in WT‐15 N compared with WT‐9 N, while there was a substantial upregulation following CASIN treatment. The results suggested that CASIN exerted a noteworthy anti‐aging effect on the proteins within Clusters 9 (Figure 4a). Further exploration of the functional enrichment cluster heatmap of GO‐CC cell components revealed that Cluster 9 was primarily enriched in ribosomes, ribosomal large subunits, ribonucleoprotein complexes, and cytoplasmic ribosomes, all of which are closely associated with ribosomal function (Figure 4b). A literature review indicated that ribosomes play a crucial role in protein synthesis as well as in the viability, growth, and proliferation of cells (Jiao et al., 2023).

A subset of 99 proteins from Cluster 9 was selected and subjected to protein expression analysis. The GO analysis revealed the involvement of ribosomes in various biological processes. The GO‐BP analysis revealed that CASIN could regulate Cdc42 signal transduction (Figure 4c). Subsequently, a subset of 11 proteins with ribosome‐related functions was selected from Cluster 9, and their expression levels were used to construct a table (Table 1). Furthermore, a protein–protein interaction (PPI) network analysis was conducted via the String database. We found that RPL4 (60S ribosomal protein L4) was the most significant difference between the WT‐15 N and WT‐9 N groups, indicating that it changed significantly with aging (Figure 4d). Moreover, the expression levels of proteins were statistically analyzed in the histogram, and RPL4 had significant statistical significance in the WT9‐N, WT9‐C, WT15‐N, and WT15‐C groups (Figure 4e). Based on the above analysis, it could be hypothesized that CASIN might play a role in skin anti‐aging through RPL4.

**TABLE 1:** List of functional differentially enriched proteins of ribosomes in Cluster9.

Protein accession	Protein description	Gene name	WT9_N	WT9_C	WT15_N	WT15_C
P14115	60S ribosomal protein L27a	RPL27A	0.65	1.13	0.68	0.66
P25444	40S ribosomal protein S2	RPS2	0.79	2.21	0.72	0.72
P35980	60S ribosomal protein L18	RPL18	0.71	1.58	0.56	0.79
P62918	60S ribosomal protein L8	RPL8	0.4	1.29	0.39	0.61
P84099	60S ribosomal protein L19	RPL19	0.97	1.26	0.7	0.84
Q8BVY0	Ribosomal L1 domain‐containing protein 1 OS = Mus musculus OX = 10,090 GN = Rsl1d1 PE = 1 SV = 1	RSL1D1	0.56	1.06	0.52	0.77
Q8BP67	60S ribosomal protein L24	RPL24	0.09	3.32	0.08	0.11
Q9CR57	60S ribosomal protein L14	RPL14	0.73	1.42	0.74	0.93
Q9CZM2	60S ribosomal protein L15	RPL15	0.84	1.2	0.67	0.76
Q9D1R9	60S ribosomal protein L34	RPL34	0.8	1.38	0.89	0.89
Q9D8E6	60S ribosomal protein L4	RPL4	0.94	1.46	0.49	0.77

### 3.5 CASIN exerts anti‐aging effects in dermal fibroblasts through RPL4 in vitro and in vivo

**FIGURE 5:**
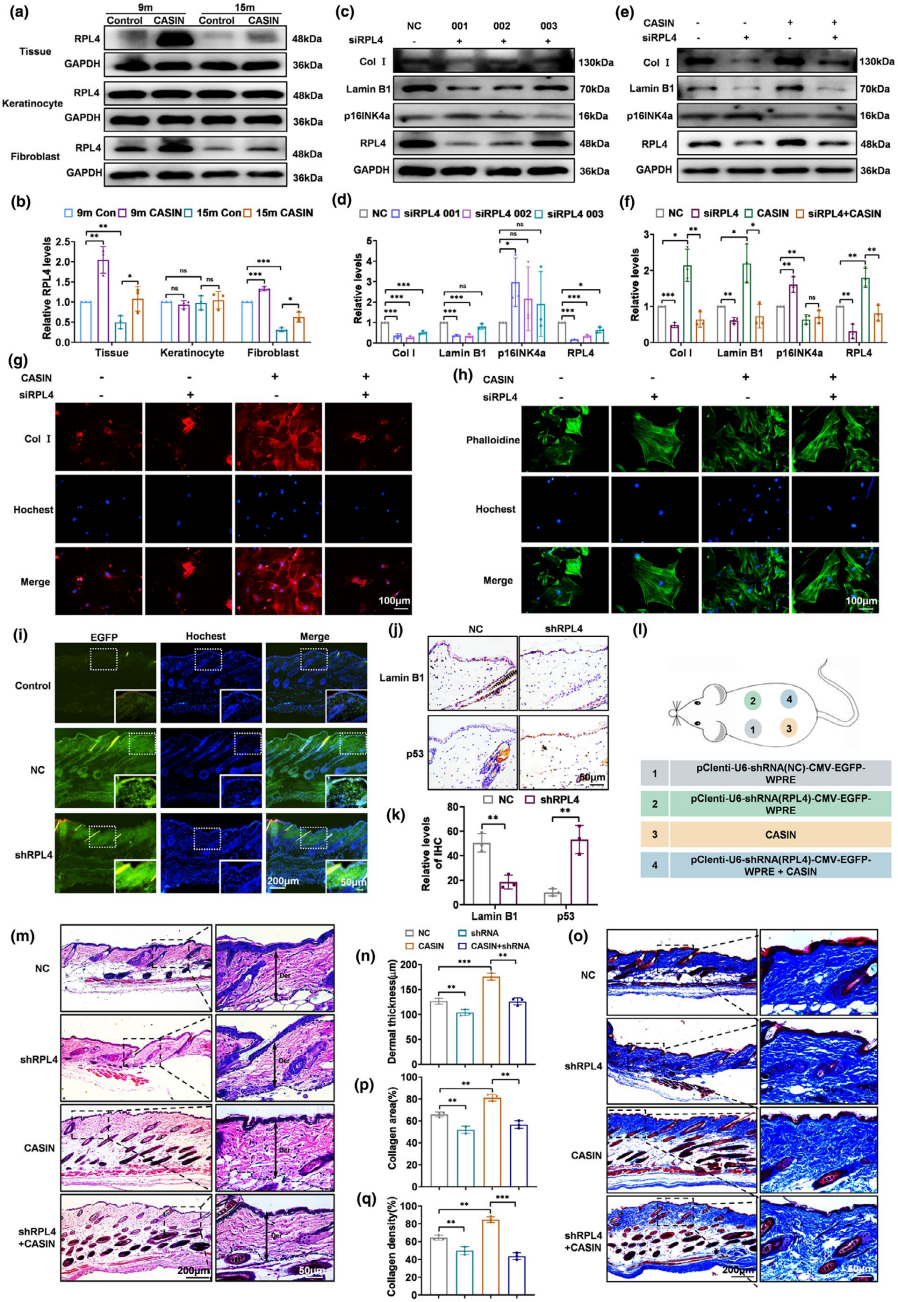
CASIN exerts anti‐aging effects in naturally aging dermics through RPL4 in vitro and in vivo. (a, b) The Western blot detection and relative expression of RPL4 in tissue, primary keratinocyte, and primary fibroblast of skin, *n* = 3. (c, d) Western blot detection of the anti‐aging effect of CASIN and relative expression of RPL4, p16INK4a, Lamin B1, and Col I after siRPL4 transfection, *n* = 3. (e, f) The Western blot detection of the anti‐aging effect of CASIN and the relative expression of RPL4, p16INK4a, Lamin B1, and Col I with the control groups NC, siRPL4‐001, CASIN, and siRPL4‐001 + CASIN. (g) Immunofluorescence staining of Col I with the control groups NC, siRPL4‐001, CASIN, and siRPL4‐001 + CASIN. (h) Phalloidin staining of primary skin fibroblasts with the control groups NC, siRPL4‐001, CASIN, and siRPL4‐001 + CASIN. (i) To evaluate the infection effect of lentivirus injection, pClenti‐U6‐shRNA (NC)‐CMV‐EGFP‐WPRE and pClenti‐U6‐shRNA (RPL4)‐CMV‐EGFP‐WPRE, both containing green fluorescent markers, were subcutaneously injected. EGFP with green fluorescent markers of lentivirus injection of frozen sections in the dermis of the Con, NC, and shRPL4 groups. (j, k) Immunohistochemical staining and statistics of relative levels of Lamin B1 and p53 with NC and shRPL4 groups, *n* = 3. (l) Animal Pattern Diagram with NC, shRPL4, CASIN, and shRPL4 + CASIN. (m, n) H&E staining and statistics of dermal thickness with NC, shRPL4, CASIN, and shRPL4 + CASIN, *n* = 3. (o‐q) MASSON staining and statistics of collagen area and collagen density with NC, shRPL4, CASIN, and shRPL4 + CASIN, *n* = 3; (scale bar = 100, 200, 50 μm). All error bars indicate SD. *p<0.05, **p<0.01, ***p<0.001.

To verify the expression of RPL4 in the epidermis and dermis, the proteins of primary keratinocytes, primary fibroblasts, and skin were extracted. The results of western blot analysis confirmed that the expression of RPL4 increased after CASIN treatment in skin and primary fibroblasts, which aligned with the outcomes of proteomic sequencing. While RPL4 remained at a constant level with or without CASIN treatment in primary keratinocytes (Figure 5a,b). The short interfering RNAs of RPL4 were transfected into primary fibroblasts; the siRPL4‐001 significantly disrupted RPL4 compared to the NC group. Following transfection with RPL4, the upregulation of the aging marker p16INK4a and the downregulation of Lamin B1 were observed, suggesting a relationship between RPL4 expression and aging. Meanwhile, col I was downregulated, suggesting a relationship between RPL4 expression and col I (Figure 5c,d). Moreover, when siRPL4‐001 transfection was combined with CASIN treatment, the levels of RPL4, Lamin B1, and p16INK4a were significantly different from the individual CASIN treatment (Figure 5e,f). The western blot and immunofluorescence staining of the expression of Col I was decreased with or without CASIN (Figure 5e,g). Meanwhile, phalloidin staining demonstrated that transfection of siRPL4‐001 resulted in an increase in volume and irregular morphology with or without CASIN (Figure 5h). In conclusion, the anti‐aging properties of CASIN were mediated by RPL4, influencing collagen synthesis and cytoskeleton morphology in fibroblasts.

Frozen section analysis demonstrated the presence of prominent green fluorescent markers in the dermis of both the NC and shRPL4 groups compared to the control group after subcutaneous injection of lentivirus, suggesting that the lentivirus successfully penetrated the dermis of the skin through subcutaneous injection on day 7 (Figure 5i). Furthermore, immunohistochemical staining results revealed significant alterations, with a decrease in Lamin B1 and an increase in p53 expression in the shRPL4 group compared to the NC group, indicating that decreased expression of RPL4 could expedite the process of skin aging (Figure 5j,k). The experimental design for the animal study is depicted in Figure 5l. Compared to NC, the administration of shRPL4 via subcutaneous injection resulted in a significant decrease in skin dermis thickness, collagen area, and density with or without CASIN treatment (Figure 5m–q). These results suggested that CASIN, through RPL4, promoted an increase in skin dermis thickness, collagen area, and collagen density, thereby exerting an anti‐aging effect.

The natural aging process is accompanied by chronic inflammation. To elucidate the roles of RPL4 and CASIN in chronic inflammation, immunohistochemical staining was performed to assess the expression levels of the pro‐inflammatory cytokine TNF‐α and the anti‐inflammatory cytokines IL‐4 and IL‐13. Immunohistochemical analysis demonstrated an age‐dependent increase in TNF‐α expression and a decrease in IL‐4 and IL‐13 expression in mice at 9, 15, and 24 months (Figure S5a–d). Furthermore, the results indicated that CASIN treatment led to a downregulation of the TNF‐α, concomitant with an upregulation of the IL‐4 and IL‐13 (Figure S5e–h). In contrast to the treatment of CASIN, immunohistochemical staining results demonstrated an increase in TNF‐α expression and a decrease in IL‐4 and IL‐13 expression in the shRPL4 group compared to the NC group (Figure S5e–h). These observations suggest that diminished expression of RPL4 promotes inflammation, corroborating our findings of reduced RPL4 expression and an enhanced inflammatory response in the skin of aging mice. Conversely, CASIN exhibits anti‐inflammatory properties, potentially contributing to the anti‐aging effects on the skin observed in these animals.

Our results revealed a hitherto undocumented function of RPL4 in fibroblasts and provide a fundamental basis for future investigations on ribosomal proteins.

### 3.6 The injection and topical application of CASIN manifest anti‐aging properties on the skin of naturally aging mice

The effective duration for CASIN injection to manifest its anti‐aging effects on the skin of naturally aging mice at 9, 15, and 24 months of age was determined using the experimental protocol illustrated in Figures S4a and S6a. The results revealed a significant increase in the thickness of the epidermis and dermis, as well as the DEJ, in the skin of mice aged 9, 15, and 24 months at days 7, 14, and 21 following subcutaneous administration of CASIN, compared to the control group (Figure S4b–i and Figure S6b–e). Additionally, findings from Masson's trichrome staining demonstrated that CASIN administration promoted collagen synthesis in the skin of naturally aging mice at the same time points. During the treatment period, a statistically significant increase in both collagen area and density was observed, along with a more compact and orderly arrangement (Figures S4j–o and Figure S6f–h). Additionally, staining of elastic fibers revealed that CASIN administration resulted in an increased quantity and thickness of elastic fibers within the dermis of naturally aging mice on days 7, 14, and 21 (Figures S4p–s and Figure S6i, j). The immunohistochemistry data demonstrated that Lamin B1 expression increased in 9‐, 15‐, and 24‐month‐old mice following CASIN therapy, as illustrated in Figures 2m,n and 3h,i; Figures S6k and S6L. It is hypothesized that the subcutaneous administration of CASIN exhibits its efficacy beginning on day 7 and continuing until day 21 in mice aged 9, 15, and 24 months.

**FIGURE 6:**
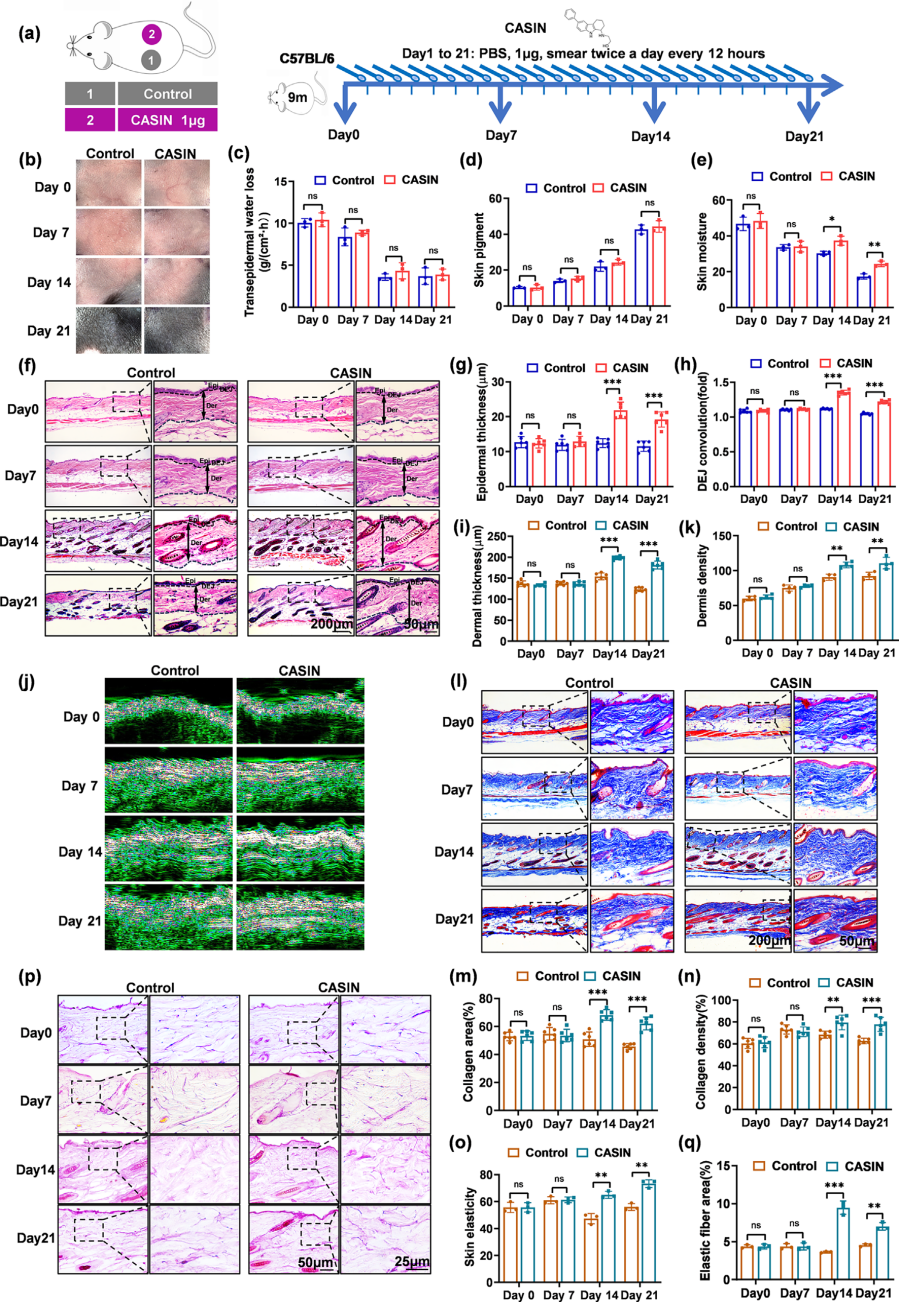
The anti‐aging effect of CASIN on the skin of naturally aging mice after continuous topical application of CASIN in the mice. (a) Animal Pattern Diagram and experimental treatment. Two evenly spaced, symmetrical circles with a diameter of 1 cm were applied to the backs of mice for 21 days. The mice were then divided into groups: Control (PBS), CASIN 1 μg. (b) Dermoscopic images. (c) Transepidermal water loss (TEWL). (d) The skin pigment. (e) The skin moisture. (f–i) H&E staining and statistics of epidermal thickness, dermal thickness, and DEJ convolution (fold) with Control and CASIN, *n* = 6. (j, k) The skin ultrasound of CASIN application and the statistics of collagen density. (l–n) MASSON staining and statistics of collagen area and collagen density with Control and CASIN, *n* = 6. (o) The skin elasticity. (p, q) Elastic fiber staining and statistics of elastic fiber area with Control and CASIN, *n* = 3; (scale bar = 200, 50, 25 μm) *p<0.05, **p<0.01, ***p<0.001.

The animal experiment scheme is depicted in Figure 6a. Based on the experimental results, no significant alterations were observed in dermoscope, transepidermal water loss (TEWL), or skin pigment following continuous smearing of CASIN for 7, 14, and 21 days (Figure 6b–d). An analysis of skin physiology indicated a significant increase in skin moisture content on days 14 and 21 after continuous CASIN treatment for 21 days, in comparison to the control group, but not on day 7 (Figure 6e). H&E staining results demonstrated a noteworthy increase in epidermal and dermal thickness and DEJ on days 14 and 21 of 9‐month‐old mice following continuous administration of CASIN (Figure 6f–i). Additionally, skin ultrasound results indicated an increase in dermal thickness on the 14th and 21st days after the application of CASIN (Figure 6j,k). Masson staining revealed a substantial growth in collagen content in the skin, with a dense and orderly arrangement on days 14 and 21, respectively (Figure 6l–n). Furthermore, the staining of elastic fibers demonstrated that the skin exhibited growth and thickening of elastic fibers on days 14 and 21 (Figure 6p,q). These findings were consistent with the measurements of skin elasticity (Figure 6o). Thus, it can be inferred from the aforementioned results that the smearing of CASIN did not yield any significant effects on day 7, whereas both the 14th and 21st days exhibited anti‐aging effects on the skin. In conclusion, CASIN might be absorbed through the skin directly by topical application, providing a simple, safe, and highly valuable application method.

## 4 DISCUSSION

Chronological skin aging leads to various dermatological issues. Intrinsic cutaneous senescence, which predominantly presents as diminished epidermal and dermal thickness and functionality, reduced collagen fiber content and density, fragmented elastic fibers, decreased skin elasticity and turgor, as well as surface roughness, contributes to the overall natural aging process (Csekes & Rackova, 2021; Kohl et al., 2011). In recent years, increased Cdc42 activity has been documented in naturally aging mice, and premature aging phenotypes have been found in multiple organs in mice with persistent Cdc42 activation. It has been reported that CASIN, a small molecule inhibitor targeting Cdc42 activity, can play an anti‐aging role (Amoah et al., 2022; Kalim et al., 2022). Our research found that CASIN enhanced epidermal and dermal thickness, enhanced DEJ, promoted growth and thickening of elastic fibers, and elevated the area and density of dermal collagen compared to the control group.

It has been observed that CASIN exhibits anti‐aging properties in various organs and tissues; however, variations in the concentration, method, and duration of CASIN treatment have been reported (Montserrat‐Vazquez et al., 2022; Tiwari et al., 2021). In this study, concentration gradients of 0.1 μg, 1 μg, and 10 μg were chosen to determine the optimal dosage for the assessment of the anti‐aging effect. All of the concentrations were kept lower than those reported in the literature to rule out potential toxicity (Florian et al., 2020; Liu et al., 2019; Wu et al., 2021). According to the results, we found that the subcutaneous injection of CASIN at 1 μg yielded a significant anti‐aging effect on the epidermis and dermis of middle‐aged and old mice. Additionally, CASIN concentrations varied among distinct cell types (Leins et al., 2018; Ubukawa et al., 2020; Wu et al., 2021). Our study revealed that CASIN, administered at a concentration of 0.5 μM, concurrently exhibited anti‐aging effects on keratinocytes and fibroblasts in the naturally aging mice. Consistent with our finding, CASIN treatment at a dose of 0.5 μM reduced Cdc42 activity in senescent MuSC, leading to enhanced division dynamics and myogenic colony capacity in senescent stem cells (Montserrat‐Vazquez et al., 2022).

It is now understood that the natural aging process is characterized by thinning of the epidermis and a decline in keratinocyte function (Lorencini et al., 2014; Wang et al., 2020). Our previous research had found that Cdc42 played a crucial role in the development of the epidermis and the formation of the epidermal barrier. The impairment of the Cdc42‐SPRR signal might be associated with skin barrier dysfunction and various skin diseases (Zhang et al., 2019). Notably, the administration of CASIN, a specific pharmacological inhibitor targeting Cdc42 activity, yielded promising results in the treatment of elderly HFSC by inhibiting the age‐related increase in Cdc42 activity and restoring the typical Wnt signal in elderly HFSC (Tiwari et al., 2021). Remarkably, the in vivo treatment of elderly mice with CASIN was found to induce hair regeneration in the skin (Tiwari et al., 2021). However, to date, there is a lack of research investigating the relationship between CASIN and keratinocytes in the skin of aging.

In the present study, we found that CASIN enhanced epidermal thickness, could enhance proliferation and differentiation, restore cytoskeletal morphology, and exhibit anti‐aging effects on primary keratinocytes in the epidermis. There were numerous studies that established the influence of Cdc42 on epidermal function. The crucial role of Cdc42 in keratinocytes for the maintenance of the skin's basement membrane has been documented (Wu et al., 2006). Furthermore, it has been demonstrated that Cdc42 plays a crucial role in maintaining the dynamic cytoskeletal polarization of keratinocytes, thereby facilitating persistent directed migration (Patwardhan et al., 2023). A study by Rohani et al. demonstrated that Cdc42 reduced the expression of MMP‐1 in keratinocytes by inhibiting ERK activity through the action of Rho GTPases (Rohani et al., 2014). Hence, it was postulated that the potential anti‐aging mechanism of CASIN in keratinocytes could potentially involve senescence‐related pathways and MAPK signaling pathways.

The aging process in the dermis of naturally aged mice is characterized by the degradation of collagen and elastic fibers, coupled with diminished fibroblast function (Heinz, 2021; Schmelzer & Duca, 2022; Wlaschek et al., 2021). Cell expansion was influenced by the multi‐domain small GTPase activating protein IQGAP1, impacting collagen remodeling through regulation of the phagocytic degradation pathway (Nakajima et al., 2021). In this study, CASIN enhanced dermal thickness, enhanced DEJ, and induced an increase in both elastic and collagen fibers in the dermis of naturally aged mice. Meanwhile, CASIN promoted the proliferation and collagen synthesis of primary fibroblasts while restoring cytoskeletal morphology and exhibiting anti‐aging effects in the dermis. However, existing research lacks insight into the relationship between CASIN and fibroblasts in the context of aging. In a study by Zhong et al., the combined action of type I collagen and fibronectin was shown to enhance glioma progression through the CDC42/AP‐1/NUPR1/Nestin signaling pathway (Zhong et al., 2021). Literature on Cdc42 and fibroblast functions indicates that P21‐activated kinase (PAK), regulated by RAC1 and Cdc42, plays a significant role. Depletion of PAK has been linked to lifespan extension and the reduction of age‐related phenotypes in mouse models with premature aging, as well as delayed aging in mammalian fibroblasts (Amirthalingam et al., 2021). Further investigation is needed to elucidate the specific mechanism through which CASIN exerts its anti‐aging effects in the dermis of naturally aged mice.

This study employed proteomic sequencing technology to investigate the mechanisms underlying the anti‐aging effects of CASIN on skin aging. Drawing on sequencing data and existing literature, we identified ribosomal proteins (RPs) as potential key players in these processes (MacInnes, 2016; Takada & Kurisaki, 2015). Meanwhile, our study presents novel evidence, both in vivo and in vitro, elucidating the involvement of ribosomal protein RPL4 in fibroblast cytoskeleton morphology and collagen synthesis. Notably, research by Luan et al. demonstrated a greater inhibitory effect on cell growth in the absence of 60S RPs compared to the depletion of 40S RP genes. Furthermore, the study revealed that deficiency in eS8/RPS8 stimulated apoptosis, while deficiency in eL13/RPL13 or eL18/RPL18 promoted cellular senescence (Luan et al., 2022). Further exploration in our study pointed to the potential significance of the ribosomal protein RPL4 (Yang et al., 2019). Literature has established RPL4's pivotal role in various cellular and tissue functions (Jongmans et al., 2018). Meanwhile, its crucial involvement in normal cell growth and proliferation, attributed to the regulation of p53 levels and p53‐dependent cell cycle arrest, has been documented (He et al., 2016). Moreover, in human B cells and FLS, RPL4 was a transcriptional regulator that regulates CD40 expression (Zou et al., 2020). Several reports have explored the association between other ribosomal proteins and fibroblasts. For example, the downregulation of ETS1 in human dermal fibroblasts (HDF) and embryonic lung fibroblasts (IMR‐90) resulted in reduced expression of ribosomal protein genes (RPGs) and a delay in cellular aging (Xiao et al., 2022). Numerous studies have indicated that chronic inflammation, driven by inflammatory cytokines, is a primary contributor to natural skin aging. Our findings indicated that CASIN exhibited anti‐inflammatory properties, while reduced expression of RPL4 was associated with increased inflammation in the skin of naturally aging mice. These results align with the observations of Zhu et al., who explored the causal relationship between ribosome levels, inflammation, and aging in human peripheral blood primary monocytes. Utilizing cycloheximide (CHX), an inhibitor that specifically binds to the 60S ribosomal subunit and impedes translation elongation, they demonstrated a marked, time‐dependent upregulation of the inflammatory cytokines IL‐6 and IL‐8 in primary monocytes (Zhu, Chen, et al., 2023). Our research, in conjunction with the findings of Zhu's study, demonstrated that a reduction in ribosome levels results in increased inflammation in both skin tissue and human peripheral blood primary monocytes. Consistent with our findings, Florian et al. observed a significant increase in the serum concentrations of the inflammatory cytokines INF‐γ, IL‐1β, and IL‐1α with aging. Furthermore, CASIN treatment of aged mice reduced the levels of these cytokines to those observed in young animals (Florian et al., 2020). Our research, in conjunction with the findings of Florian's study, demonstrated the role of CASIN in mediating anti‐inflammatory effects during the natural aging process, particularly in the context of skin and blood cells. Our results unveil a novel function of RPL4 in skin aging, providing a foundational basis for future investigations into ribosomal proteins.

According to relevant literature, compounds with a molecular weight below 500 Daltons can permeate the skin barrier and be absorbed through the skin (Bos & Meinardi, 2000). CASIN, with a molecular weight of 306.4, falls within this range, suggesting its potential for transdermal absorption through non‐invasive application. The study aimed to evaluate the anti‐aging effects of CASIN on the dorsal skin of mice using two different approaches: subcutaneous injection and non‐invasive application. Results from subcutaneous injection were observed after 7 days, while noticeable effects from topical application were evident after 14 days. Both interventions demonstrated sustained effects for a duration of 21 days. Indeed, subcutaneous injection offers faster absorption and action but involves a procedure that can cause trauma and requires a high level of technical expertise from the operator. In contrast, CASIN could be absorbed directly through the skin via smearing, providing a simple, safe, and highly valuable application method. These findings highlight that CASIN, as a small‐molecule inhibitor, exhibits the advantageous feature of easy transdermal absorption, allowing it to effectively manifest its anti‐aging effects.

## 5 CONCLUSION

To summarize, our investigation establishes that CASIN induces anti‐aging effects on the dorsal skin of naturally aging mice aged 9, 15, and 24 months. CASIN promotes the proliferation, differentiation, and colony formation of epidermal keratinocytes, restoring their cytoskeletal morphology in naturally aging mice. Moreover, CASIN exhibits the capability to enhance collagen synthesis and restore skeletal morphology in fibroblasts, facilitated by the ribosomal protein RPL4 using proteomic sequencing. This leads to an increase in dermal thickness and collagen density, ultimately contributing to its anti‐aging properties. Subcutaneous injection demonstrates efficacy by day 7, while topical application shows efficacy by the 14th day, with both methods maintaining their effects for up to 21 days. Meanwhile, CASIN exhibited anti‐inflammatory properties, while reduced expression of RPL4 was associated with increased inflammation in the skin of naturally aging mice. These findings suggest a promising potential for CASIN as an effective intervention in anti‐aging skin care. Our results unveil a novel function of RPL4 in skin aging, providing a foundational basis for future investigations into ribosomal proteins.

## AUTHOR CONTRIBUTIONS

Y.Z. and X.W: experimental design, data collection, data analysis and interpretation, and article writing; J.H., X.Z., L.B., Y.Z., F.L., S.W. and M.Z: data collection; Lu Z. and Lin Z: conception and design, financial support, article writing, and final approval of the article. All authors have read and agreed to the published version of the manuscript.

## CONFLICT OF INTEREST STATEMENT

The authors declared that they had no known competing financial interests or personal relationships that could have appeared to influence the work reported in this paper.

## Supporting information


Figure S1.
Figure S2.
FigurE S3.
Figure S4.
Figure S5.
Figure S6.

## Data Availability

The datasets used and/or analyzed during the current study are available from the corresponding author on reasonable request.
