# Supplementary material for: CASIN exerts anti‐aging effects through RPL4 on the skin of naturally aging mice
Source: Aging Cell. 2024 Sep 17;23(12):e14333. doi: 10.1111/acel.14333 (PMC11634736; doi:10.1111/acel.14333)
Supplement: Supplementary file 1 — Figure S1. Figure S2. FigurE S3. Figure S4. Figure S5. Figure S6. [file ACEL-23-e14333-s001.docx]

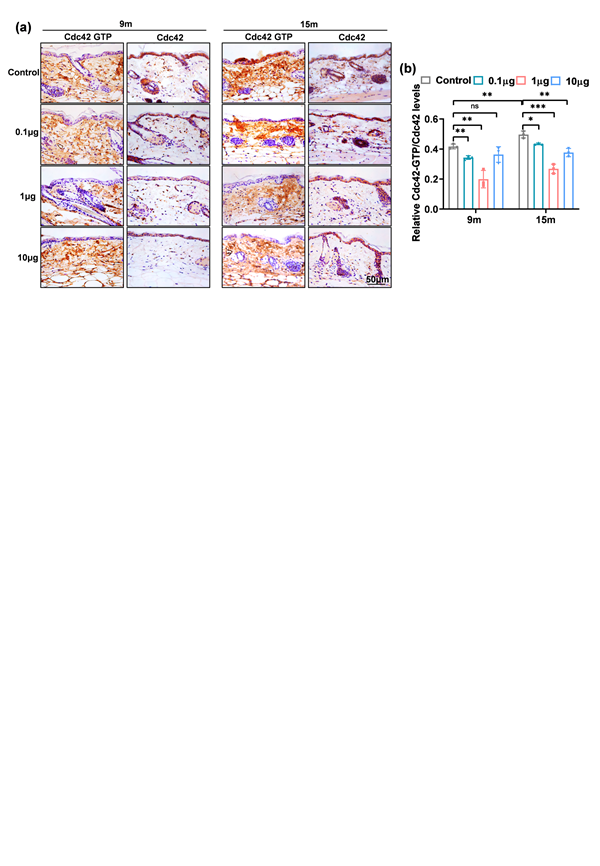


FIGURE S1 The expression of Cdc42 GTP/Cdc42 decreased in both 9-month-old and 15-month-old mice following CASIN therapy. (a, b), Immunohistochemical staining and statistics of relative levels of Cdc42 GTP/Cdc42 expression in the skin of naturally aging mice at 9 and 15 months; n=3; (scale bar=50μm). All error bars indicate SD. **P*＜0.05, ***P*＜0.01, ****P*＜0.001.


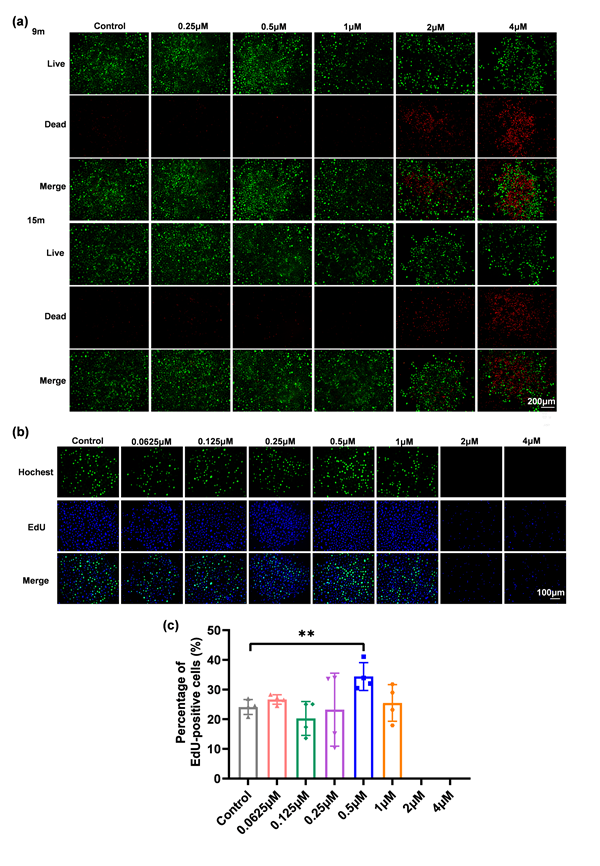


FIGURE S2 The optimal concentration of CASIN for its anti-aging effect in primary keratinocytes of naturally aging mouse skin. (a), Cell death staining showed CASIN treatment of 4μM, 2μM, 1μM, 0.5μM and 0.25μM for 24h of primary keratinocytes. (b, c), Images of EdU positive proliferating cells and statistics of the proliferation rate of 4μM, 2μM, 1μM, 0.5μM, 0.25μM, 0.125μM and 0.0625μM of primary keratinocytes, n=4; (scale bar=200, 100μm), **P*＜0.05, ***P*＜0.01, ****P*＜0.001.


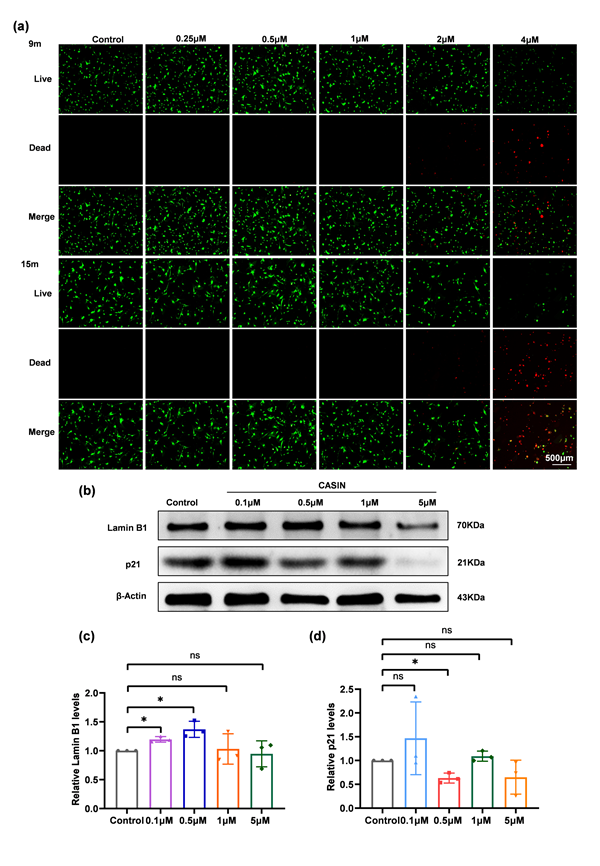


FIGURE S3 The optimal concentration of CASIN for its anti-aging effect in primary fibroblasts of naturally aging mouse skin. (a), Cell death staining showed CASIN treatment of 4μM, 2μM, 1μM, 0.5μM and 0.25μM for 24h of primary fibroblasts. (b-d), Western blot of the anti-aging effect of CASIN and relative expression of LaminB1 and p21 with the group of 5μM, 1μM, 0.5μM and 0.1μM of primary fibroblasts, n=3; (scale bar=500μm), **P*＜0.05, ***P*＜0.01, ****P*＜0.001.


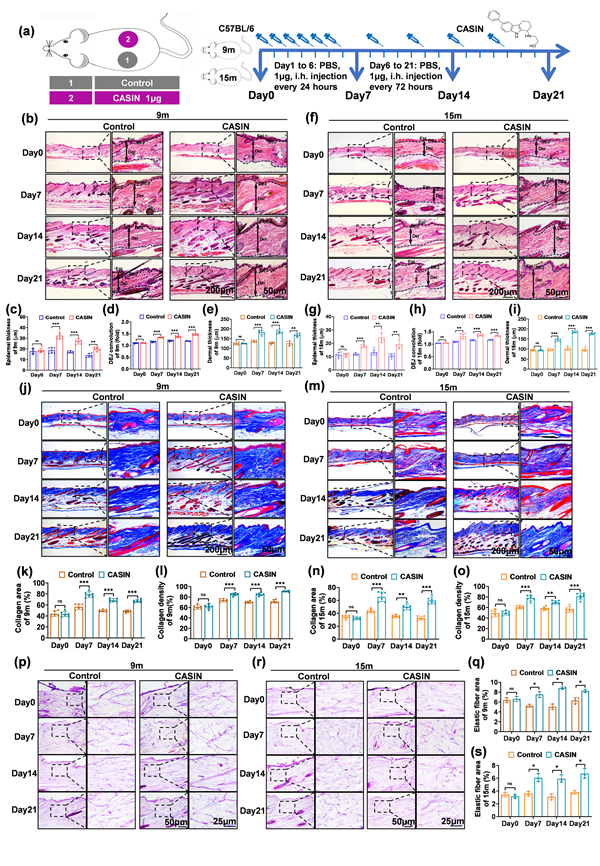


FIGURE S4 Effective time of CASIN subcutaneous injection for anti-aging effect in naturally aging mouse skin on days 7, 14, and 21 in the mice at 9 and 15 months. (a) Animal Pattern Diagram and experimental treatment. Two evenly spaced, symmetrical circles with a diameter of 1cm were applied to the backs of mice for 21 days. The mice were then divided into groups: Control (PBS), CASIN 1μg. (b-i) HE staining and statistics of epidermal thickness, DEJ convolution(fold), and dermal thickness, with Control and CASIN, n = 6. (j-o) MASSON staining and statistics of collagen area and collagen density with Control and CASIN, n = 6. (p-s) Elastic fibers staining and statistics of elastic fibers area with Control and CASIN, n = 3; (scale bar = 200, 50, 25μm). All error bars indicate SD. **P*＜0.05, ***P*＜0.01, ****P*＜0.001.


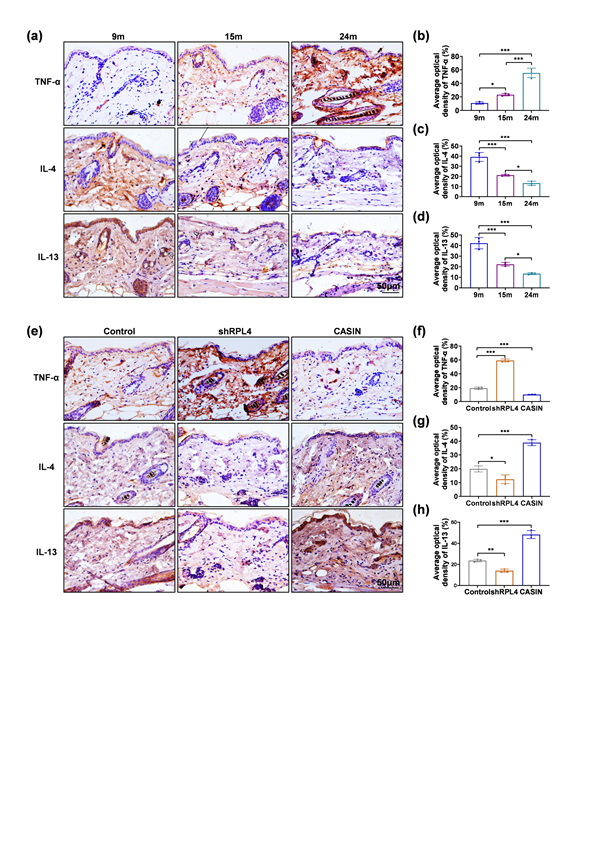


FIGURE S5 CASIN and RPL4 affect inflammatory factors in naturally aging mice. (a-d) Immunohistochemical staining and statistics of relative levels of TNF-α , IL-4 and IL-13 in the mice at 9, 15 and 24 months, n = 3. (e-h) Immunohistochemical staining and statistics of relative levels of TNF-α, IL-4 and IL-13 with Control, shRPL4, CASIN, n = 3; (scale bar = 50μm). All error bars indicate SD. **P*＜0.05, ***P*＜0.01, ****P*＜0.001.


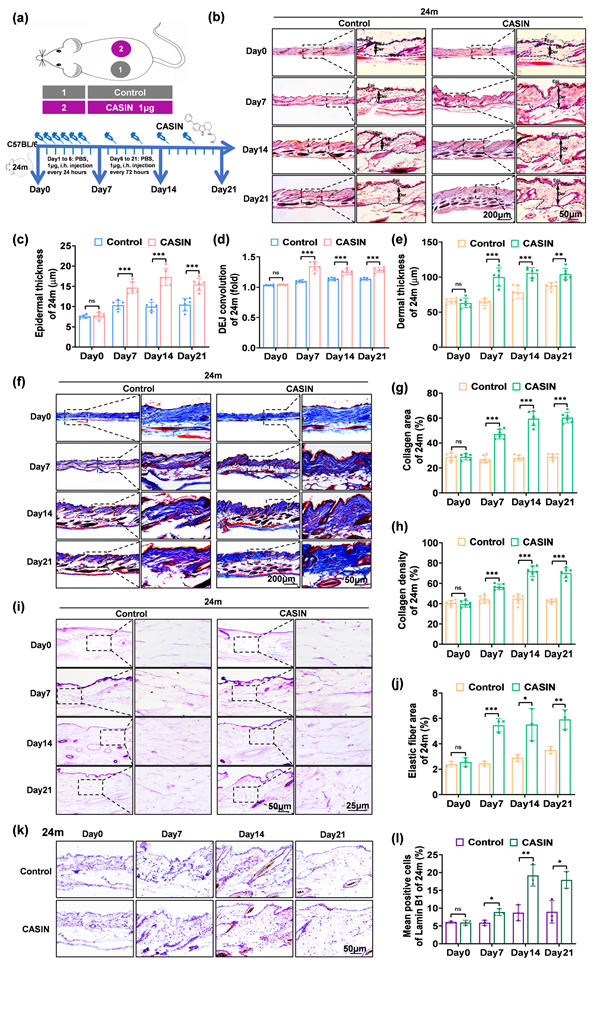


FIGURE S6 Effective time of CASIN subcutaneous injection for anti-aging effect in naturally aging mouse skin on days 7, 14, and 21 in the mice at 24 months. (a) Animal Pattern Diagram and experimental treatment. Two evenly spaced, symmetrical circles with a diameter of 1cm were applied to the backs of mice for 21 days. The mice were then divided into groups: Control (PBS), CASIN 1μg. (b-e) HE staining and statistics of epidermal thickness, DEJ convolution(fold), and dermal thickness, with Control and CASIN, n = 6. (f-h) MASSON staining and statistics of collagen area and collagen density with Control and CASIN, n = 6. (i, j) Elastic fibers staining and statistics of elastic fibers area with Control and CASIN, n = 3; (k, l) Immunohistochemical staining and statistics of relative levels of Lamin B1, n=3. (scale bar = 200, 50, 25μm). All error bars indicate SD. **P*＜0.05, ***P*＜0.01, ****P*＜0.001.
